# Differentiation of *Escherichia coli* and *Shigella flexneri* by Metabolite Profiles Obtained Using Gold Nanoparticles-Based Surface-Assisted Laser Desorption/Ionization Mass Spectrometry

**DOI:** 10.3390/pathogens14010019

**Published:** 2024-12-30

**Authors:** Adrian Arendowski

**Affiliations:** 1 Department of Inorganic and Analytical Chemistry, Faculty of Chemistry, Rzeszów University of Technology, Powstańców Warszawy 6, 35-959 Rzeszów, Poland; a.arendowski@prz.edu.pl; Tel.: +48-17-865-1541; 2 Centre for Modern Interdisciplinary Technologies, Nicolaus Copernicus University, Wileńska 4, 87-100 Toruń, Poland

**Keywords:** gold nanoparticles, identification, mass spectrometry, metabolites, microorganisms, surface-assisted laser desorption/ionization

## Abstract

*Escherichia coli* and *Shigella flexneri* are challenging to differentiate using methods such as phenotyping, 16S rRNA sequencing, or protein profiling through matrix-assisted laser desorption/ionization mass spectrometry (MALDI MS) due to their close relatedness. This study explores the potential for identifying *E. coli* and *S. flexneri* by incorporating reference spectra of metabolite profiles, obtained via surface-assisted laser desorption/ionization mass spectrometry (SALDI MS) employing gold nanoparticles (AuNPs), into the Bruker Biotyper database. Metabolite extracts from *E. coli* and *S. flexneri* cells were prepared using liquid–liquid extraction in a chloroform–methanol–water system. The extracts were analyzed using Au-SALDI MS in positive ion mode, and reference spectra, compiled from 30 spectra for each bacterium, were added to the database. Identification of bacteria based on metabolite fingerprints in the Biotyper database produced correct results with scores exceeding 2.75. The results of Partial Least Squares-Discriminant Analysis (PLS-DA) demonstrated that the metabolomic approach could accurately differentiate the microorganisms under study. A panel of nine *m*/*z* values was also identified, each with an area under the ROC curve of above 0.8, enabling accurate identification of *E. coli* and *S. flexneri*. A search of metabolite databases allowed the following compounds to be assigned to the selected *m*/*z* values: *N*-acetylputrescine, arginine, 2-maleylacetate, benzoyl phosphate, *N*8-acetylspermidine, alanyl-glutamate, 4-hydroxy-2,3,4,5-tetrahydrodipicolinate, and sucrose. The analyses showed that identification of bacteria based on metabolite profiles obtained by the Au-SALDI MS method is feasible and can be useful for distinguishing closely related microorganisms that are difficult to differentiate by other techniques.

## 1. Introduction

*Escherichia coli* and *Shigella flexneri* are strongly associated Gram-negative bacteria classified within the Enterobacterales family [1,2], which exhibit distinct epidemiological and clinical characteristics [3]. *S. flexneri* is a significant enteric pathogen, being the most important cause of dysentery in Asia and Africa [4], whereas *E. coli* strains in the human gut are generally commensal [5,6]. Due to many common phenotypic features and high nucleotide sequence similarity [1], differentiating these bacteria presents a diagnostic challenge [2]. Sophisticated microbial identification methods, such as 16S rDNA sequencing and proteomic analysis via matrix-assisted laser desorption/ionization-time-of-flight mass spectrometry (MALDI-TOF MS), are widely used. However, these techniques often struggle to reliably differentiate *S. flexneri* from *E. coli* [3]. Rapid and accurate microorganism identification is crucial for clinical diagnosis and treatment [7]. For this reason, many researchers have attempted to use various analytical methods to distinguish between these microorganisms. Feng et al. used Fourier transform infrared spectroscopy (FT-IR) and multivariate analysis [8], while Liu and co-workers used surface-enhanced Raman spectroscopy (SERS) with silver nanoparticles and machine learning to classify *E. coli* and *Shigella* strains [9]. A different approach was used by Elena-Herrmann’s group, which used nuclear magnetic resonance (NMR) spectroscopy to study the exometabolome of bacteria in culture media and then statistical analysis of the results to discriminate between *E. coli* and *Shigella* spp. [10]. Li et al. used a biochemical approach to identify *E. coli* and *Shigella* species based on the presence on MALDI MS spectra of signals from specific decomposition products of lysine and ornithine by bacterial cellular enzymes, achieving a correct identification rate of 97% [11]. The approach employed by Li et al. can be described as targeted metabolomics. Another type of analysis is the so-called untargeted metabolomics, which focuses on recording metabolomic profiles or metabolic fingerprints. This method allows for the characterization of a specific phenotype based on all metabolites present in a cell at a given time and under specific conditions [12]. Comparing metabolic profiles facilitates the identification of compounds that differentiate organisms or cells depending on variable environmental conditions. Such an approach is applied, among other things, in the identification of cancer biomarkers. The MALDI MS technique was also used to analyze the lipid profiles of bacteria and distinguish between *E. coli* and *Shigella* spp. based on them [6,13]. However, due to the presence of a high chemical background from the matrix peaks in the mass spectral range below *m*/*z* 700, MALDI MS is not the most suitable technique for measuring low-molecular-weight (LMW) compounds, including metabolites and lipids [14]. A method that could be more useful for this type of analysis is surface-assisted laser desorption/ionization mass spectrometry (SALDI MS), which uses various types of nanoparticles and nanostructures to ionize compounds [15]. This avoids the presence of matrix signals in the low-mass region interfering with the spectrum, as well as the occurrence of the “sweet spot” phenomenon, i.e., inhomogeneous co-crystallization of the analyte and matrix. Gold nanostructures are among the most widely used nanomaterials in laser desorption/ionization mass spectrometry. Gold nanoparticles (AuNPs) exhibit a very high absorption coefficient for radiation above 400 nm, which is attributed to their large specific surface area. Due to their chemical inertness and biocompatibility, gold can be employed in biological studies without affecting the analyte molecules. The synthesis of gold nanostructures is relatively straightforward, and there are numerous possibilities for surface modification [16]. One solution among the many gold-based SALDI techniques described, which allows for the analysis of LMW compounds of different polarity, including from complex biological matrices, is a gold nanoparticle-enhanced target (AuNPET) [17]. This method has already been used to analyze the metabolic profiles of molds and gave the possibility to identify significantly more compounds compared to the classical MALDI MS [18]. In addition, mass spectrometry methods including SALDI may also be more suitable for use in the metabolic fingerprint analysis of bacteria than methods such as NMR or FT-IR proposed by other authors [8,10]. Metabolic fingerprints are complex mixtures of compounds of biological origin that are often present in low concentrations. NMR and FT-IR have lower sensitivity compared to MS techniques, and interpretation of spectra of mixtures of metabolites obtained by these methods can be difficult.

The aim of this study was to investigate the possibility of distinguishing closely related *E. coli* and *S. flexneri* bacteria on the basis of metabolite profiles obtained by the gold nanoparticle-based SALDI MS technique. For this purpose, MS spectra of metabolic extracts from *E. coli* and *S. flexneri* were performed on an AuNPET plate. The signals recorded on the spectra were analyzed using statistical tools and added to a commercial database—Bruker Biotyper—creating reference spectra for bacterial identification.

## 2. Material and Methods

### 2.1. Reagents and Materials

The bacterial strains, *Shigella flexneri* ATCC^®^ 12022™ and *Escherichia coli* ATCC^®^ 11775™, were bought on KWIK-STIK™ from Argenta (Poznań, Poland). Nutrient Agar was obtained from NEOGEN (Heywood, UK). Bacterial Test Standard (BTS) and α-cyano-4-hydroxycinnamic acid (CHCA) were bought from Bruker Daltonics GmbH (Bremen, Germany). Chloro(trimethylphosphite)gold(I) (97+% purity), pyridine–borane complex (BH_3_:py) at a ~8 M borane concentration, and trifluoroacetic acid (analytical standard) were purchased from Sigma-Aldrich (St. Louis, MO, USA). Water, acetonitrile, and methanol (HPLC grade) and sodium chloride (pure p.a. grade) were obtained from Honeywell (Seelze, Germany). Formic acid and chloroform (pure p.a. grade) were bought from CHEMPUR (Piekary Śląskie, Poland). A custom-made plate of stainless steel, grade H17, with dimensions of 75 × 25 × 0.8 mm was locally manufactured using waterjet cutting and was used with the Bruker Daltonics MTP Slide Adapter II (Bremen, Germany).

### 2.2. MALDI Matrix and Calibrant Preparation

The CHCA matrix was formulated as a 10 mg/mL solution using a mixture of acetonitrile and 0.1% trifluoroacetic acid in water, with a volume ratio of 30:70. Bruker BTS MALDI calibrant was prepared by dissolving the contents of the tube in 50 µL of a mixture consisting of 50% acetonitrile, 47.5% water, and 2.5% trifluoroacetic acid.

### 2.3. Preparation of AuNPs-Coated SALDI Target

A SALDI target covered with gold nanoparticles was prepared following a method similar to that described by Sekuła et al. [19]. A total of 25 mg of chloro(trimethylphosphite)gold(I) and 50 mL of acetonitrile were added to a glass beaker and left on an ultrasonic bath for 5 min. An H17-grade stainless-steel plate measuring 75 × 25 × 0.8 mm was placed in a 100 × 15 mm glass Petri dish and flooded with a solution of chloro(trimethylphosphite)gold(I) in acetonitrile followed by the addition of 173 µL of 8 M BH3:py complex in pyridine. After a 48 h reaction period, the gold-coated plate was washed several times with acetonitrile, wiped with a cotton wool ball, and washed three more times with hot water and acetonitrile.

### 2.4. Bacterial Cultures

*Escherichia coli* ATCC^®^ 11775™ and *Shigella flexneri* ATCC^®^ 12022™ were cultured on Nutrient Agar at 37 °C for 24 h, with each bacterium grown in 36 replicates. The biomass from each Petri dish was collected and suspended in 20 mL of sterile saline to a density of 5 McFarland. The suspensions were then centrifuged for 10 min at 3000 rpm, after which the supernatant was discarded. The grown bacterial colonies were analyzed using the standard proteomic approach through the MALDI-TOF MS method, employing a Bruker microflex LT mass spectrometer with the Biotyper database (MBT Compass Library Revision H; Bruker Daltonics GmbH, Bremen, Germany). Protein extracts were prepared following standard protocols by transferring a loop of biomass onto an MSP 96 polished steel target spot (Bruker Daltonics GmbH, Bremen, Germany). This was followed by the addition of 1 µL of 70% formic acid and 1 µL of CHCA matrix. MS spectra were calibrated using BTS as an external calibrator and a quadratic calibration strategy. The protein-based MALDI-TOF MS spectra are shown in Supplementary Materials (Figure S1), and bacterial identification results are presented in Supplementary Materials (Table S1).

### 2.5. Metabolite Extraction

Metabolite extracts from the bacteria were prepared using the Bligh and Dyer method [20]—1 mL of methanol and 1 mL of chloroform were added to the centrifuged biomass; then, the samples were left in an ultrasonic bath for 10 min, after which 900 µL of water was added to each and vortexed for 10 min. To accelerate phase separation, samples were centrifuged at 2000 rpm for 10 min at 4 °C. The upper (water) phase was collected in a separate centrifuge tube and used in the SALDI MS experiment.

### 2.6. SALDI MS Analysis of Metabolite Extracts

**Figure 1:**
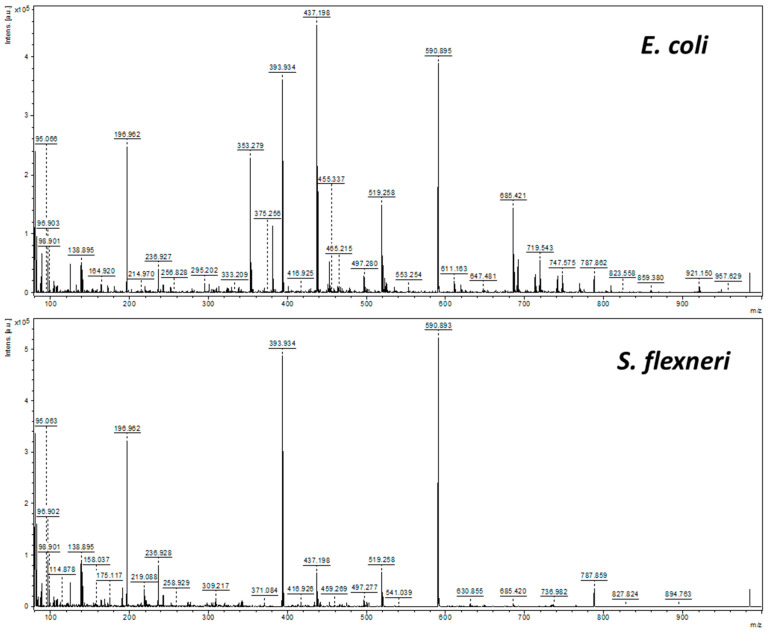
Gold nanoparticle-based SALDI MS spectrum of metabolite extract from *E. coli* (**top**) and *S. flexneri* (**bottom**).

Measurements were conducted using a Bruker ultrafleXtreme time-of-flight mass spectrometer (Bruker Daltonics GmbH, Bremen, Germany) in reflector positive ion mode within the *m*/*z* range of 80 to 1000. The mass spectrometer was equipped with a neodymium-doped yttrium–aluminum garnet (Nd:YAG) SmartBeam II laser operating at a wavelength of 355 nm and a maximum frequency of 2000 Hz. The laser was working at 60% power, and the laser pulse energy was approximately 100–190 µJ. The spectrometer was controlled using FlexControl 3.3 software, and data processing was performed using FlexAnalysis 3.3 (Bruker Daltonics GmbH). The deflection value was set to ions of *m*/*z* lower than 75. The first accelerating voltage was 25.07 kV, and the second ion-source voltage was held at 22.41 kV. The reflector voltages accounted for 26.64 (first) and 13.59 kV (second). The value of detector gain for the reflector was 4× (2010 V). Additionally, 0.5 µL of lipid extract samples was spotted onto the AuNPs-coated steel plate. A sum of four spectra of 1000 laser shots each was taken for all samples. Laser shots were made with the application of the default random walk. Mass calibration was performed using an internal standard (gold ions and clusters from Au^+^ to Au_5_^+^) with a quadratic calibration strategy. Example spectra are shown in Figure 1.

### 2.7. Data Analysis

Modifying the standard methods implemented in the MALDI Biotyper Compass Explorer database (Bruker Daltonics GmbH), an MSP (Main Spectra Profile) reference spectrum was created from 30 calibrated MS spectra for each bacterium. In the preprocessing method, the lower bound was set at 80, and the upper bound was set at 1000. In the MSP creation method, the desired peak frequency parameter was set at 50%, the and max. desired peak number for the MSP was set at 200. The list of signals entered into the Biotyper database for *E. coli* is presented in Supplementary Materials (Table S2) and for *S. flexneri* in Supplementary Materials (Table S3). Mass spectra for metabolite extracts from cultures of each bacterium conducted in 6 Petri dishes were treated as spectra of unknown samples for database testing.

The MS data were subjected to statistical analysis using MetaboAnalyst 6.0 software [21]. The data were normalized using the cube-root-transformed sum and subjected to default Pareto scaling. To construct the receiver operating characteristic (ROC) curve, random forests were selected as the classification method, and RandomForest was used for feature ranking.

## 3. Results and Discussion

The basis for using the MALDI TOF MS technique to identify microorganisms was defined in the 1990s, when papers were published demonstrating the utility of protein profiles to differentiate bacteria [22,23,24,25]. Identification of microorganisms by MALDI is based on the analysis of protein extracts or whole bacterial cells using a CHCA matrix in the *m*/*z* range of 2–20 kDa and comparison of the resulting spectrum with reference spectra contained in the database [26]. The Bruker Biotyper database was the first commercially available system for identifying microorganisms using MALDI MS. It is a well-established database, widely used in diagnostic laboratories, which now includes protein fingerprint spectra for more than 4000 species of microorganisms in its library [27]. Software that compares mass spectra of microorganisms calculates a scoring value based on the similarities between the spectra recorded for the sample and those deposited in the database. The score value indicates the correctness of the spectra match and, thus, the assignment of the sample to a particular species. In the case of the Bruker Biotyper database, the score takes values from 0 to 3, with a value above 2.0 considered a valid identification at the species level. In contrast, values between 2.0 and 1.7 represent reliable identifications at the genus level [28].

The Biotyper database allows the creation of additional reference spectra for new microorganisms. Taking advantage of this possibility, an attempt was made to add spectra of metabolic profiles obtained by the Au-SALDI TOF MS technique for *E. coli* and *S. flexneri* by modifying the manufacturer’s standard methods. Signals from cellular metabolites appear on MS spectra, mainly in the *m*/*z* range below 1000 [29]. For this reason, in the method of preprocessing the spectra, the lower limit of *m*/*z* was set at 80, and the upper limit was set at 1000. The AuNPET method was chosen for metabolite analyses, which allows the analysis of LMW compounds without a chemical background [19]. In the investigated range, only five signals from gold clusters appear on the MS spectra: Au^+^ (*m*/*z* 196.966), Au_2_^+^ (*m*/*z* 393.933), Au_3_^+^ (*m*/*z* 590.899), Au_4_^+^ (*m*/*z* 787.866), and Au_5_^+^ (*m*/*z* 984.832) [30], which at the same time enable accurate internal calibration of the spectra.

Thirty calibrated Au-SALDI MS spectra obtained for metabolic extracts from *E. coli* and *S. flexneri* (Figure 1) were uploaded to the Bruker Biotyper database, after which reference MSP spectra were created using the modified method described above. The lists of signals present in the generated MSP spectra are described in Supplementary Materials (Figure S1 and Table S1). In addition to peaks originating from gold, the lists mainly contained signals below *m*/*z* 700. This means that the signals with the highest intensities and those repeated in at least 50% of the spectra occurred in the *m*/*z* 80–700 range. This may seemingly conflict with the definition of metabolomics studies, in which metabolites are defined as small molecules with masses up to 1500 Da [31]. However, this definition often includes lipids as a specific group of metabolites. Analyzing the *E. coli* Metabolome Database (ECMDB) [32] reveals that more than half of the compounds included there have masses below 700 Da, and most of the compounds with higher masses are lipids. Using liquid–liquid extraction with immiscible polar and nonpolar phases, it can be assumed that due to their structure, most of the lipids went to the nonpolar phase, which was not analyzed in this study. Hence, the observed *m*/*z* range of signals present in the spectra seems to be most suitable for analyzing bacterial metabolites and further attempts to identify microorganisms based on metabolic profiles.

**Figure 2:**
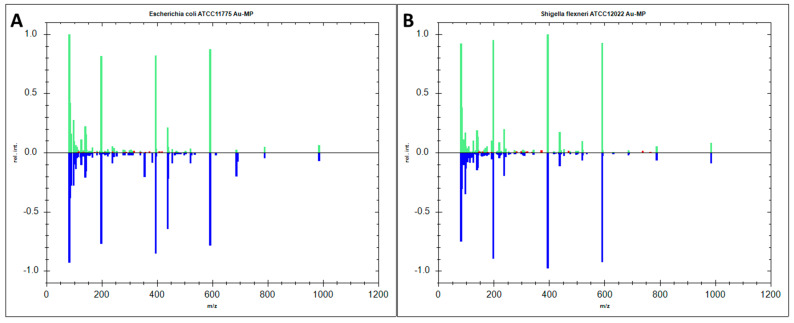
The matching test (top) and reference (bottom) spectra obtained by AuNPs-SALDI MS for metabolite extracts from *E. coli* (**A**) and *S. flexneri* (**B**) in the Bruker Biotyper software (MBT Compass Library Revision H). The top spectrum—the spectrum of the test sample, the bottom spectrum—the reference spectrum.

Six AuNPs-SALDI MS spectra from metabolic extracts of *E. coli* and *S. flexneri* were used as test samples to evaluate the performance of the newly created database. The test sample spectra were calibrated in the same manner as the reference spectra, and their alignment was conducted using Bruker Biotyper software (MBT Compass Library Revision H) (Figure 2). The comparison between the test and reference spectra stored in the database resulted in 100% accurate species identification for both bacterial species. The score values for *E. coli* and for *S. flexneri* were over 2.75, which in the Bruker Biotyper software indicates a high degree of confidence in identification (Table 1). However, for the *E. coli* test samples, the second-best matched species was *S. flexneri* with scores above 2.70, while for the *S. flexneri* samples it was *E. coli* with scores above 2.60. This means that, despite a correct match, there is a risk of confusion due to the small differences in the scores obtained between the bacterial species analyzed. Moreover, the analyzed species are phenotypically and genotypically similar, potentially leading to the production of comparable metabolites. Bunge et al. also noted similarities in the volatile metabolites produced by *E. coli* and *S. flexneri* [33], as did Rautureau et al. in the metabolic footprint of both bacteria [10]. Especially considering that the bacteria were cultured under identical conditions, it is not surprising that no distinctive signals were detected as in the work of Li et al. [11]. However, in contrast to a commercial database based on protein spectra in the *m*/*z* 2–20 kDa range, it was possible to correctly identify both bacteria using the research protocol proposed in this study (Table 1 and Table S1). The result obtained using the classical method of bacterial identification on the MALDI MS instrument, in which all protein profile spectra were classified as *E. coli*, does not differ from the observations of other authors [2,3]. The protein profiles analyzed by the MALDI MS technique are mainly composed of evolutionarily conserved ribosomal proteins, resulting in difficulties in reliably distinguishing between closely related microbial species such as *E. coli* and *S. flexneri* [34]. However, a model built using artificial neural networks allowed for the correct differentiation of *E. coli* from *Shigella* species based on protein profiles obtained by the MALDI MS technique [35]. The approach used by Everley et al. [36] to analyze the proteome of *E. coli* and *Shigella* spp. by LC-ESI-MS could also provide some solution to this problem. This technique, compared to MALDI MS, showed several additional signals above 15 kDa, thus enabling distinction between these closely related microorganisms. Nevertheless, it has not been implemented on a wider scale in laboratory practice.

**Figure 3:**
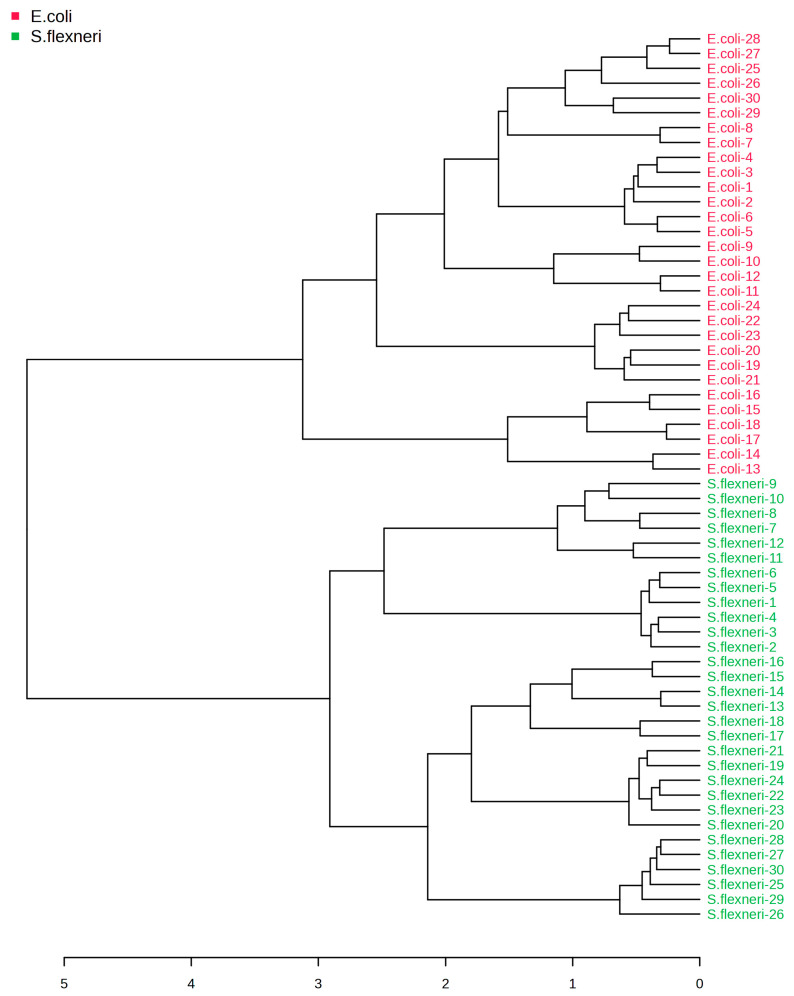
Dendrogram for Au-SALDI MS data from *E. coli* and *S. flexneri* metabolite extracts.

**Figure 4:**
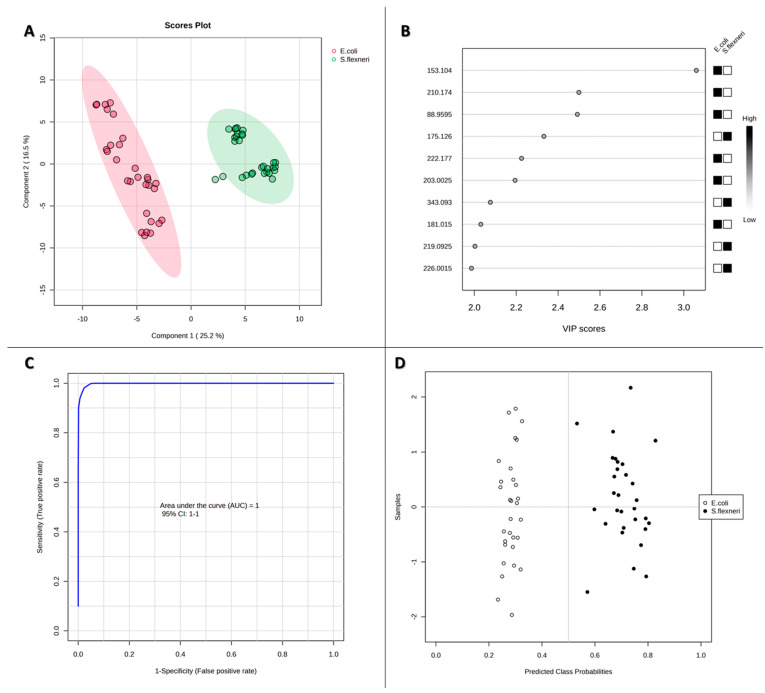
The graphical representation of the statistical analysis of AuNPs-SALDI MS data from metabolite extracts of *E. coli* and *S. flexneri* includes the following: PLS-DA component 1 versus component 2 (**A**), PLS-DA VIP scores (**B**), multivariate ROC analysis (**C**), and predicted class probabilities derived from cross-validation (**D**).

In addition to the analysis of the results using the Bruker Biotyper database, statistical analysis was carried out in the online software MetaboAnalyst 6.0 [21] for Au-SALDI MS spectra of bacterial metabolic profiles. A hierarchical clustering dendrogram was plotted in the software using the average clustering algorithm and the Pearson distance measure (Figure 3). The dendrogram shows complete separation of the groups into *E. coli* and *S. flexneri*; this indicates correct clustering of the metabolic extract spectra relative to the bacterial species studied. A similar situation is observed in the 2D plot obtained by Partial Least Squares-Discriminant Analysis (PLS-DA) (Figure 4A) and K-means Clustering based on Principal Component Analysis (PCA) (Supplementary Materials Figure S2). When a model based on the random forest method was used to classify MS data, it was possible to achieve error-free matching of spectra to the species of bacteria analyzed (Supplementary Materials Figure S3). All three methods used allowed the bacteria to be completely separated into two groups. This means that the applied statistical analysis of peaks with signal-to-noise ratios above 3 from Au-SALDI MS spectra of metabolic extracts of *E. coli* and *S. flexneri* allows for the correct differentiation of the microorganisms studied and that, despite similar phenotypic and genotypic characteristics, these bacteria present different metabolic profiles. Previous work using gold nanoparticles for SALDI MS analysis of metabolites from molds and statistical analysis of the results [18] already suggested the possibility of using this methodology to discriminate between microorganisms, but this has not yet been investigated for closely related bacterial species. Similarly, spectra obtained in low mass ranges using SALDI methods based on silver nanoparticles allowed for the effective classification of selected microorganisms with an overall accuracy of 75–100% [37], as well as distinguishing between sensitive and cefotaxime-resistant *E. coli* strains [38].

**Figure 5:**
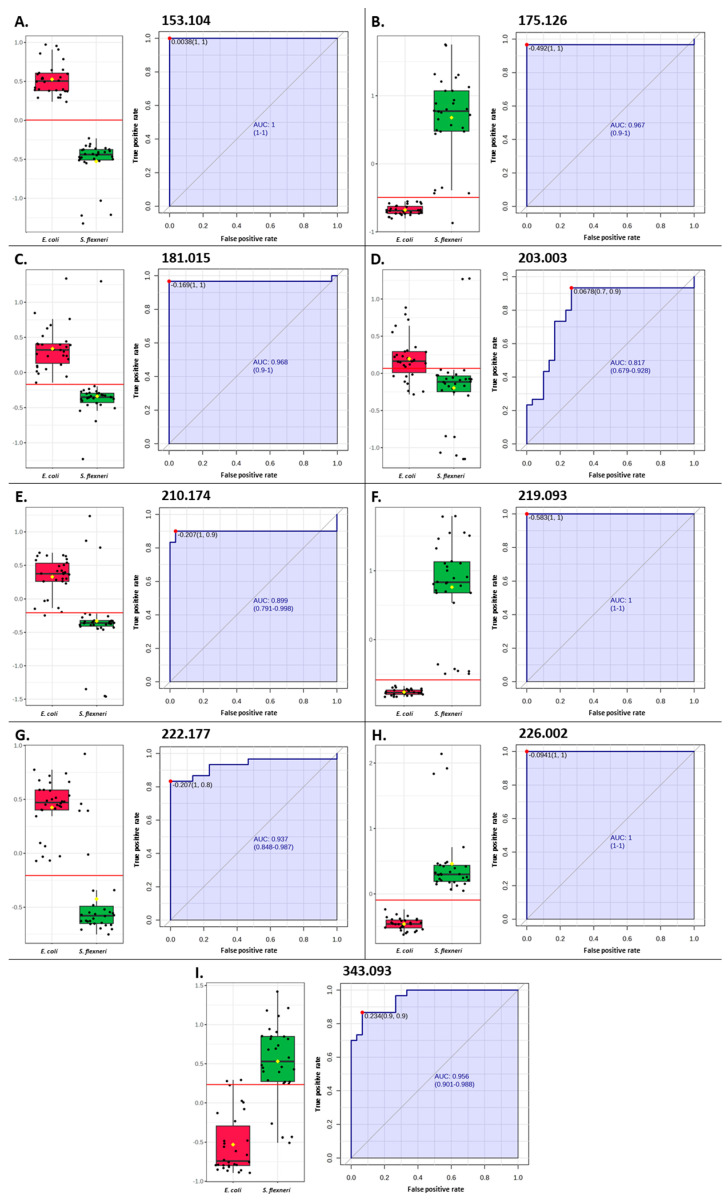
Box plots and ROC analysis of nine *m*/*z* values most differentiating between *E. coli* and *S. flexneri:* 153.104 (**A**), 175.126 (**B**), 181.015 (**C**), 203.003 (**D**) 210.174 (**E**), 219.093 (**F**), 222.177 (**G**), 226.002 (**H**), and 343.093 (**I**).

Based on the analysis of the results obtained by the PLS-DA method for MS data from metabolic extracts from *E. coli* and *S. flexneri*, ten *m*/*z* values were selected whose variable significance in projection (VIP) was at least 2 (Figure 4B), meaning that they had a significant effect on the separation of bacteria into two groups. These were the following *m*/*z* values: 175.126, 219.0925, 226.0015, and 343.093 (with higher intensities in the MS spectra of *S. flexneri* extracts) and 88.9595, 153.104, 181.015, 203.0025, 210.174, and 222.177 (with higher intensities in the MS spectra of *E. coli* extracts). Subsequently, for all values, a receiver operating characteristic (ROC) curve was plotted, illustrating the sensitivity of the given model—i.e., the proportion of true positive results relative to specificity, which represents the proportion of false positive results. For nine features, values for the area under the curve (AUC) above 0.8 were obtained (Figure 5); only for the *m*/*z* equal to 88.9595 was the value lower; therefore, this signal was excluded from further analysis. The AUC cutoff value of 0.8 was determined from the literature to provide good test accuracy [39]. Analysis of the correctness of the classification of the samples (Supplementary Materials Figures S4–S12) showed that individual *m*/*z* values correctly classify 78 to 100% of the samples. For the nine *m*/*z* values selected, the *E. coli* Metabolome Database [32] was searched to match possible bacterial metabolites. The putative identification allowed us to assign compounds to the eight *m*/*z* values with an error of less than 0.03 *m*/*z*, and all compound information was collected in Table 2. Metabolites assigned to *m*/*z* values belonged to such groups of compounds as amino acids, dipeptides and their derivatives, polyamines, organic acids, and carbohydrates (Table 2). All of the matched compounds have previously been described as occurring in microorganisms. They are natural products or substrates of the metabolic pathways of *E. coli* and similar bacteria. Only for the *m*/*z* value of 222.177 could no metabolite be assigned. It could be a fragment of some larger molecule or a compound that has not yet been detected in bacteria. The HMDB database [40] matched this *m*/*z* value with a compound called dodecanamide, also known as lauramide, and it belongs to a class of organic compounds known as fatty amides. Multivariate ROC analysis based on the PLS-DA method was also performed for a panel of nine selected differential values (Figure 4C). This yielded a 100% correct classification of all samples (Figure 4D), and the AUC value was 1, indicating that the selected *m*/*z* values could be biomarkers for distinguishing between the tested bacteria. This appears to be a significant achievement, as some studies show that the metabolites of *E. coli* and *Shigella* spp. form a single collection [41].

In summary, the method presented here is only a prelude to research into the wider application of metabolic fingerprinting for microbial identification. The research protocol presented here could be used on a wider scale in the future to create a more comprehensive database based on bacterial metabolic profiles, which could be integrated into existing diagnostic processes, especially for microorganisms that cannot be distinguished by existing methods. Additionally, the method has the potential to be used in typing strains involved in the spread of nosocomial infections. However, this method requires further research and testing due to the possible misclassification of bacteria due to slight differences between the scores obtained (Table 1). Perhaps optimization of the metabolite extraction protocol would allow for more differential results.

## 4. Conclusions

The findings reported in this study indicate that the metabolic fingerprint of bacteria, obtained through the Au-SALDI MS technique, holds potential for identifying microorganisms, particularly those species that are not distinguishable by conventional methods. Integrating reference spectra of bacterial metabolic profiles into the Bruker Biotyper database and automating the technique suggests that the approach proposed here could eventually be developed into a commercially viable protocol for use in diagnostic laboratories. This research serves as a proof of concept, demonstrating the effectiveness of the metabolic approach in differentiating microorganisms. However, further investigations and an expansion of the metabolic fingerprint database to include additional species and strains are necessary to fully realize its potential.

## Figures and Tables

**Table 1:** Results of Au-SALDI MS classification of microorganisms based on metabolite profiles.

Analyte Name	Organism (Best Match)	Score Value	Organism (Second Best Match)	Score Value
*E. coli* sample 1	*Escherichia coli*	2.81	*Shigella flexneri*	2.74
*E. coli* sample 2	*Escherichia coli*	2.79	*Shigella flexneri*	2.71
*E. coli* sample 3	*Escherichia coli*	2.78	*Shigella flexneri*	2.70
*E. coli* sample 4	*Escherichia coli*	2.79	*Shigella flexneri*	2.72
*E. coli* sample 5	*Escherichia coli*	2.80	*Shigella flexneri*	2.74
*E. coli* sample 6	*Escherichia coli*	2.78	*Shigella flexneri*	2.71
*S. flexneri* sample 1	*Shigella flexneri*	2.87	*Escherichia coli*	2.68
*S. flexneri* sample 2	*Shigella flexneri*	2.81	*Escherichia coli*	2.65
*S. flexneri* sample 3	*Shigella flexneri*	2.78	*Escherichia coli*	2.64
*S. flexneri* sample 4	*Shigella flexneri*	2.79	*Escherichia coli*	2.65
*S. flexneri* sample 5	*Shigella flexneri*	2.86	*Escherichia coli*	2.68
*S. flexneri* sample 6	*Shigella flexneri*	2.82	*Escherichia coli*	2.65

**Table 2:** List of ions and compounds found by statistical analysis of Au-SALDI MS spectra of metabolic extracts of *E. coli* and *S. flexneri*.

Experimental *m*/*z*	Calculated *m*/*z*	Δ*m*/*z*	Ion Formula	Metabolite ^a^	ECMDB ^b^ Number	Regulation ^c^		VIP ^d^	AUC ^e^	Accuracy [%]	Figure ^f^
						*E. coli*	*S. flexneri*				
153.104	153.100	0.004	[C_6_H_14_N_2_O+Na]^+^	*N*-Acetylputrescine	ECMDB02064	↑	↓	3.06	1	100	5A, S4
175.126	175.119	0.007	[C_6_H_14_N_4_O_2_+H]^+^	L-Arginine	ECMDB00517	↓	↑	2.33	0.967	91.67	5B, S5
181.015	181.011	0.004	[C_6_H_6_O_5_+Na]^+^	2-Maleylacetate	ECMDB20045	↑	↓	2.03	0.968	95	5C, S6
203.003	203.010	0.007	[C_7_H_7_O_5_P+H]^+^	Benzoyl phosphate	ECMDB20121	↑	↓	2.19	0.817	78.33	5D, S7
210.174	210.158	0.016	[C_9_H_21_N_3_O+Na]^+^	*N*8-Acetylspermidine	ECMDB21254	↑	↓	2.50	0.899	85	5E, S8
219.093	219.098	0.005	[C_8_H_14_N_2_O_5_+H]^+^	L-Alanyl-D-glutamate	ECMDB21241	↓	↑	2.01	1	100	5F, S9
222.177	-	-	-	-	-	↑	↓	2.23	0.937	86.67	5G, S10
226.002	226.011	0.009	[C_7_H_9_NO_5_+K]^+^	(*2S,4S*)-4-Hydroxy-2,3,4,5-tetrahydrodipicolinate	ECMDB23778	↓	↑	2.00	1	100	5H, S11
343.093	343.123	0.03	[C_12_H_22_O_11_+H]^+^	Sucrose	ECMDB00258	↓	↑	2.07	0.956	88.33	5I, S12

^a^ Putative identification. ^b^ *E. coli* Metabolome Database. ^c^ Regulation of the intensity in samples, ↑ intensity up-regulation, ↓ intensity down-regulation. ^d^ VIP score obtained on the basis of PLS-DA analysis. ^e^ Area under the ROC curve. ^f^ Figures S4–S12 can be found in the Supplementary Materials.

## Data Availability

The research data presented in this paper are available in the RepOD repository (https://doi.org/10.18150/XGMFKO, accessed on 12 December 2024).

## Supplementary Materials

The following supporting information can be downloaded at: https://www.mdpi.com/article/10.3390/pathogens14010019/s1, Figure S1: MALDI MS spectrum of protein extract from *E. coli* (A) and *S. flexneri* (B); Table S1: Results of MALDI MS classification of microorganisms based on protein profiles; Table S2: List of peaks entering the Biotyper database for metabolite extracts from *Escherichia coli* ATCC® 11775™; Table S3: List of peaks entering the Biotyper database for metabolite extracts from *Shigella flexneri* ATCC® 12022™; Figure S2: K-means clustering based on principal component analysis (PCA) for Au-SALDI MS data from *E. coli* and *S. flexneri* metabolite extracts; Figure S3: Random Forest classification for Au-SALDI MS data from *E. coli* and *S. flexneri* metabolite extracts; Figure S4: Predicted class probabilities for *m*/*z* 153.104 obtained for Au-SALDI MS data from *E. coli* and *S. flexneri* metabolite extracts; Figure S5: Predicted class probabilities for *m*/*z* 175.126 obtained for Au-SALDI MS data from *E. coli* and *S. flexneri* metabolite extracts; Figure S6: Predicted class probabilities for *m*/*z* 181.015 obtained for Au-SALDI MS data from *E. coli* and *S. flexneri* metabolite extracts; Figure S7: Predicted class probabilities for *m*/*z* 203.003 obtained for Au-SALDI MS data from *E. coli* and S. flexneri metabolite extracts; Figure S8: Predicted class probabilities for *m*/*z* 210.174 obtained for Au-SALDI MS data from *E. coli* and *S. flexneri* metabolite extracts; Figure S9: Predicted class probabilities for *m*/*z* 219.093 obtained for Au-SALDI MS data from *E. coli* and *S. flexneri* metabolite extracts; Figure S10: Predicted class probabilities for *m*/*z* 222.177 obtained for Au-SALDI MS data from *E. coli* and *S. flexneri* metabolite extracts; Figure S11: Predicted class probabilities for *m*/*z* 226.002 obtained for Au-SALDI MS data from *E. coli* and *S. flexneri* metabolite extracts; Figure S12: Predicted class probabilities for *m*/*z* 343.093 obtained for Au-SALDI MS data from *E. coli* and *S. flexneri* metabolite extracts.
