# Supplementary material for: Differentiation of Escherichia coli and Shigella flexneri by Metabolite Profiles Obtained Using Gold Nanoparticles-Based Surface-Assisted Laser Desorption/Ionization Mass Spectrometry
Source: Pathogens. 2024 Dec 30;14(1):19. doi: 10.3390/pathogens14010019 (PMC11768538; doi:10.3390/pathogens14010019)
Supplement: Supplementary file 1 [file pathogens-14-00019-s001.zip › pathogens-3385399-supplementary.pdf]

Supplementary Materials

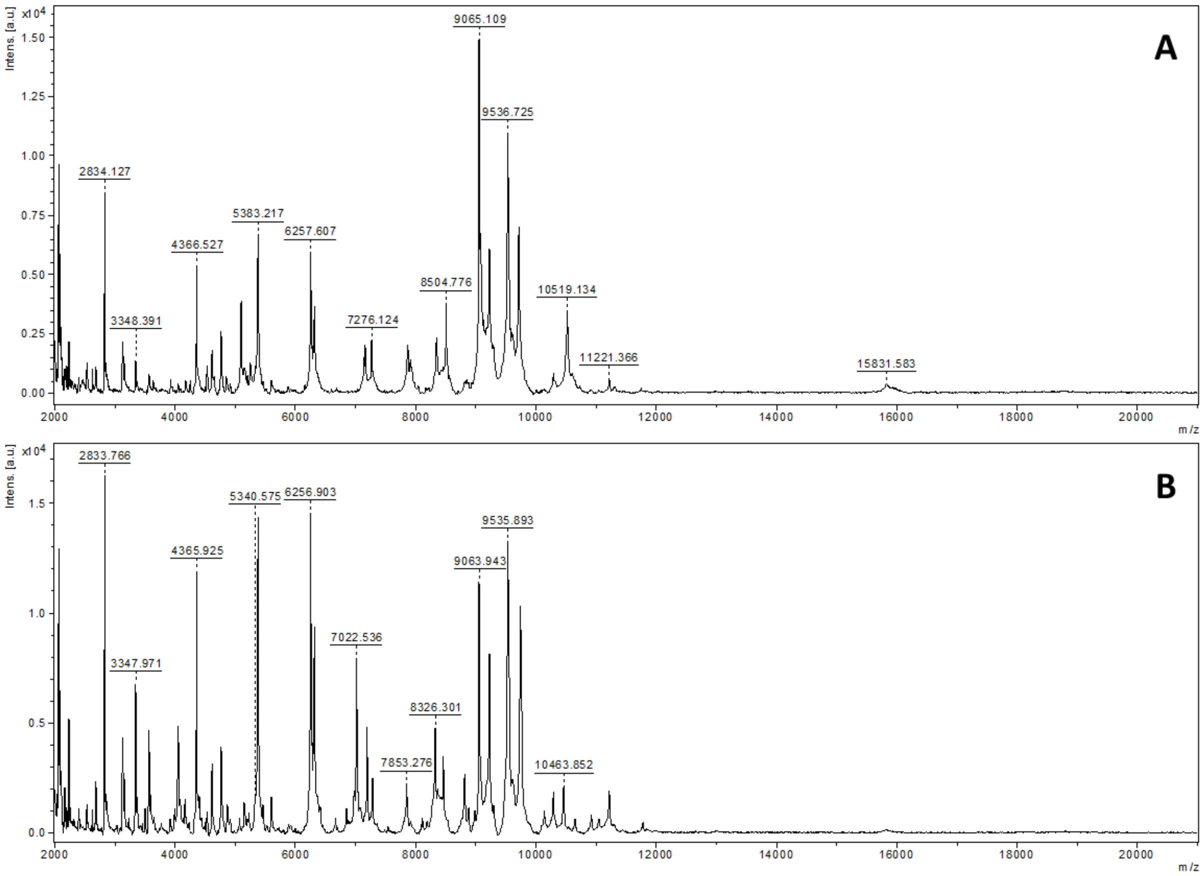

Figure S1. MALDI MS spectrum of protein extract from *E. coli* (**A**) and *S. flexneri* (**B**).

Table S1. Results of MALDI MS classification of microorganisms based on protein profiles.

Analyte Name	Organism (best match)	Score Value	Organism (second best match)	Score Value
E. coli 1	*Escherichia coli*	2.26	*Escherichia coli*	2.21
E. coli 2	*Escherichia coli*	2.24	*Escherichia coli*	2.23
E. coli 3	*Escherichia coli*	2.22	*Escherichia coli*	2.03
E. coli 4	*Escherichia coli*	2.20	*Escherichia coli*	2.07
E. coli 5	*Escherichia coli*	2.22	*Escherichia coli*	2.17
E. coli 6	*Escherichia coli*	2.17	*Escherichia coli*	2.15
S. flexneri 1	*Escherichia coli*	2.06	*Escherichia coli*	2.03
S. flexneri 2	*Escherichia coli*	2.07	*Escherichia coli*	2.03
S. flexneri 3	*Escherichia coli*	2.13	*Escherichia coli*	2.03
S. flexneri 4	*Escherichia coli*	2.05	*Escherichia coli*	2.00
S. flexneri 5	*Escherichia coli*	2.08	*Escherichia coli*	2.07
S. flexneri 6	*Escherichia coli*	2.12	*Escherichia coli*	2.05

Table S2. List of peaks entering the Biotyper database for metabolite extracts from *Escherichia coli* ATCC® 11775™

m/z [Da]	Intensity [%]	Weight [%]	Frequency [%]	In Peak Lists
80.93	92.93	100	100	30
81.02	2.99	100	100	30
81.33	1.59	100	80	24
82.93	38.44	100	100	30
83.02	1.44	100	66.7	20
84.06	1.87	100	80	24
87.96	11.14	100	100	30
88.97	27.74	100	100	30
96.91	27.82	100	100	30
98.91	9.87	100	100	30
103.95	4.49	100	100	30
104.95	13.73	100	100	30
106.90	3.44	100	100	30
108.91	4.59	100	100	30
112.89	3.62	100	86.7	26
120.93	4.11	100	100	30
122.93	2.27	100	100	30
124.93	10.16	100	100	30
133.07	2.96	100	100	30
135.07	1.41	100	50	15
138.91	21.07	100	100	30
140.08	3.37	100	100	30
140.91	15.80	100	100	30
142.92	2.49	100	100	30
146.94	1.89	100	86.7	26
153.11	2.59	100	100	30
154.89	2.41	100	83.3	25

156.05	1.44	100	50	15
156.89	2.20	100	80	24
159.03	1.30	100	63.3	19
164.93	3.90	100	100	30
180.99	2.00	100	100	30
191.09	1.80	100	83.3	25
196.98	76.71	100	100	30
198.88	1.19	100	66.7	20
203.01	1.12	100	63.3	19
210.18	2.28	100	80	24
214.99	1.47	100	90	27
219.97	3.10	100	100	30
220.97	1.46	100	60	18
225.01	1.17	100	60	18
236.95	8.89	100	100	30
242.97	3.48	100	100	30
252.20	3.08	100	100	30
252.92	1.31	100	83.3	25
277.17	1.93	100	80	24
282.21	2.33	100	93.3	28
295.22	2.16	100	56.7	17
299.15	2.11	100	80	24
303.91	2.15	100	100	30
305.90	1.68	100	93.3	28
309.24	1.51	100	93.3	28
310.25	1.95	100	93.3	28
338.28	2.33	100	83.3	25
353.31	20.57	100	73.3	22
354.30	4.88	100	66.7	20
381.34	8.60	100	73.3	22
393.97	84.83	100	100	30
394.97	2.91	100	100	30
437.23	64.42	100	100	30
438.23	22.08	100	100	30
439.24	4.66	100	100	30
441.34	1.18	100	63.3	19
453.22	9.04	100	100	30
454.22	2.26	100	96.7	29
463.23	2.10	100	100	30
469.23	1.11	100	60	18
474.92	1.19	100	60	18
479.24	1.35	100	60	18
485.21	1.29	100	50	15
497.31	1.90	100	63.3	19
500.87	1.20	100	60	18
502.87	1.14	100	60	18
519.30	9.02	100	100	30
520.31	2.84	100	100	30
521.15	2.19	100	80	24
523.15	2.18	100	80	24
535.29	1.54	100	53.3	16
590.94	78.11	100	93.3	28
591.17	1.55	100	93.3	28

592.03	1.68	100	63.3	19
611.21	2.33	100	53.3	16
685.47	19.98	100	100	30
686.47	9.08	100	100	30
687.47	2.07	100	100	30
691.57	7.48	100	56.7	17
787.91	4.76	100	100	30
984.89	6.74	100	100	30

Table S3. List of peaks entering the Biotyper database for metabolite extracts from *Shigella flexneri* ATCC® 12022™

m/z **[Da]**	**Intensity [%]**	**Weight [%]**	**Frequency [%]**	**In Peak Lists**
80.93	74.87	100	100	30
81.02	2.39	100	80	24
81.33	1.35	100	80	24
82.04	0.91	100	66.7	20
82.93	30.74	100	100	30
83.02	1.29	100	73.3	22
84.06	3.84	100	100	30
87.96	8.10	100	100	30
88.97	10.48	100	100	30
96.91	35.04	100	100	30
98.91	12.98	100	100	30
103.94	4.45	100	100	30
104.95	7.77	100	100	30
106.90	3.79	100	100	30
108.91	4.67	100	100	30
112.89	8.51	100	100	30
114.89	4.54	100	83.3	25
119.93	2.28	100	60	18
120.93	4.07	100	100	30
122.94	1.56	100	100	30
124.93	8.21	100	100	30
128.07	1.00	100	73.3	22
133.06	1.26	100	83.3	25
136.04	1.44	100	100	30
137.03	0.90	100	73.3	22
138.05	0.96	100	53.3	16
138.91	14.44	100	100	30
140.07	3.11	100	80	24
140.91	12.51	100	100	30
142.92	2.01	100	100	30
154.89	2.60	100	100	30
156.04	1.34	100	70	21
156.89	3.01	100	100	30
158.92	0.83	100	53.3	16
164.93	2.71	100	100	30
169.11	2.28	100	80	24
172.88	1.30	100	80	24
175.12	2.69	100	83.3	25

180.91	1.68	100	70	21
191.09	5.29	100	80	24
196.98	89.60	100	100	30
197.12	1.59	100	100	30
197.60	1.02	100	80	24
211.00	1.16	100	93.3	28
214.02	0.90	100	93.3	28
214.99	1.81	100	100	30
219.10	4.39	100	80	24
219.97	3.18	100	100	30
220.97	2.48	100	100	30
222.99	1.29	100	100	30
223.99	1.21	100	100	30
225.00	1.51	100	100	30
235.07	0.95	100	50	15
235.95	1.30	100	60	18
236.94	19.53	100	100	30
239.00	1.22	100	100	30
240.02	0.79	100	63.3	19
240.97	0.96	100	86.7	26
242.97	3.44	100	100	30
252.92	1.78	100	100	30
258.95	1.24	100	60	18
274.14	1.37	100	80	24
275.07	0.95	100	60	18
277.16	1.27	100	80	24
298.16	1.32	100	56.7	17
303.91	2.26	100	100	30
305.90	1.72	100	100	30
309.24	2.53	100	100	30
310.24	0.84	100	56.7	17
342.14	1.54	100	63.3	19
343.11	1.58	100	80	24
393.33	1.83	100	96.7	29
393.96	97.57	100	100	30
394.14	1.26	100	86.7	26
394.97	4.91	100	100	30
416.95	1.13	100	93.3	28
422.97	0.88	100	56.7	17
433.92	1.30	100	76.7	23
437.23	11.33	100	100	30
438.23	3.29	100	90	27
441.33	1.16	100	100	30
453.21	2.55	100	83.3	25
474.92	0.99	100	60	18
497.30	1.40	100	96.7	29
500.86	1.27	100	100	30
502.87	1.22	100	100	30
519.29	6.29	100	100	30
520.30	1.92	100	100	30
535.27	1.08	100	60	18
590.93	92.43	100	100	30
591.14	2.27	100	93.3	28

592.02	2.56	100	100	30
630.90	1.30	100	73.3	22
685.46	1.93	100	93.3	28
686.46	1.21	100	50	15
787.90	6.64	100	100	30
984.88	8.95	100	100	30

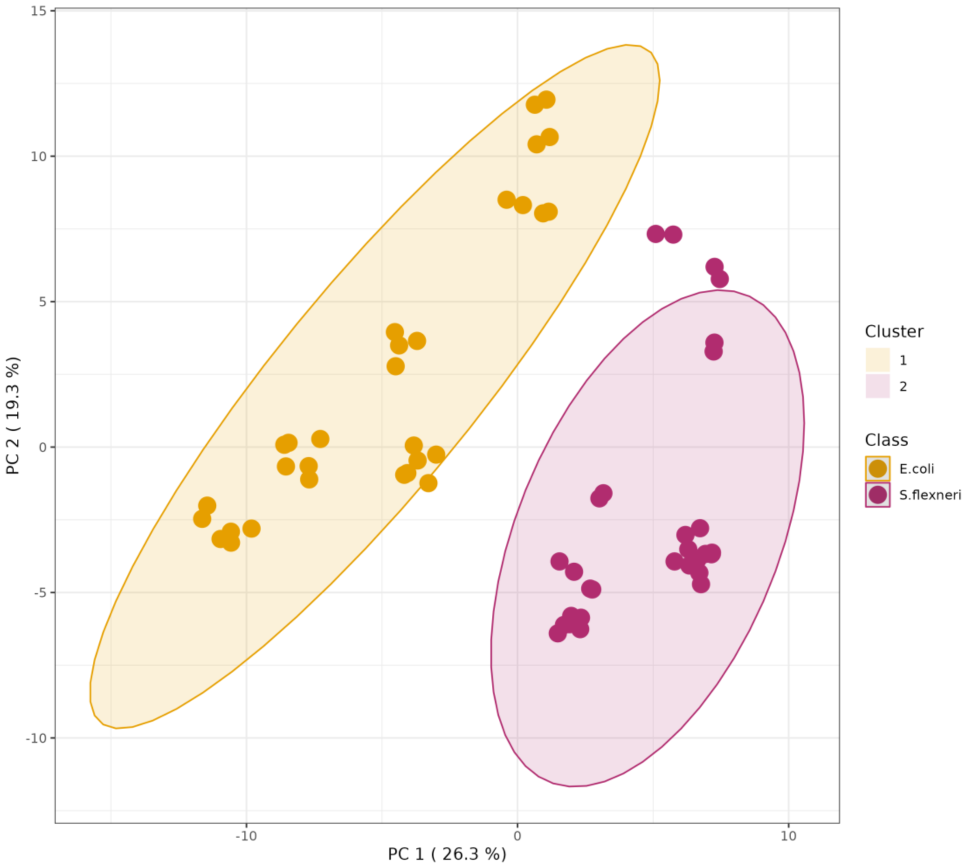

Figure S2. K-means clustering based on principal component analysis (PCA) for Au-SALDI MS data from *E. coli* and *S. flexneri* metabolite extracts.

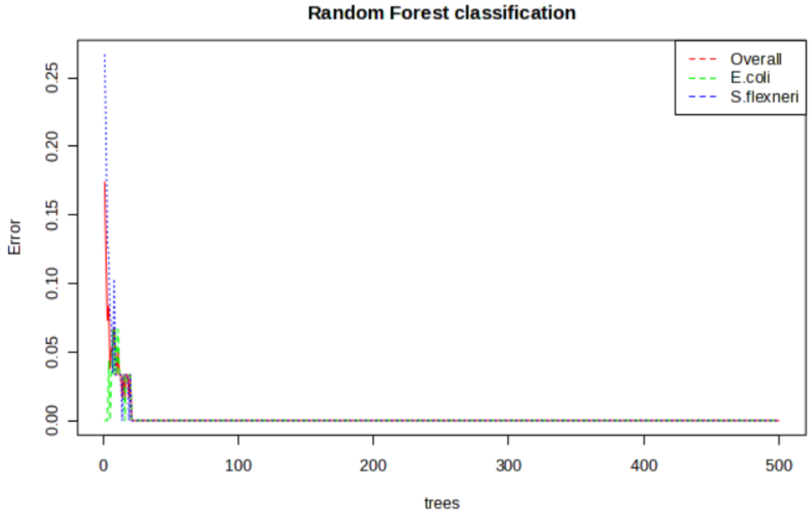

The OOB error is 0.0

	E.coli	S.flexneri	class.error
E.coli	30	0	0.0
S.flexneri	0	30	0.0

Figure S3. Random Forest classification for Au-SALDI MS data from *E. coli* and *S. flexneri* metabolite extracts.

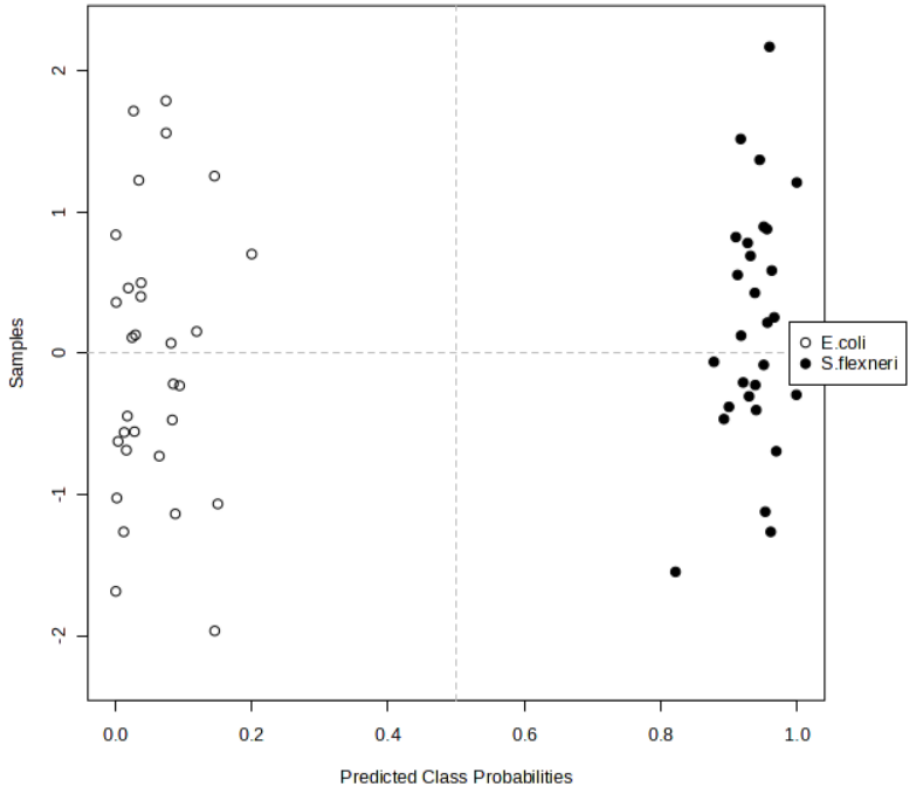

Confusion Matrix
(Cross-Validation)

	0	1
0	30	0
1	0	30

Figure S4. Predicted class probabilities for *m/z* 153.104 obtained for Au-SALDI MS data from *E. coli* and *S. flexneri* metabolite extracts.

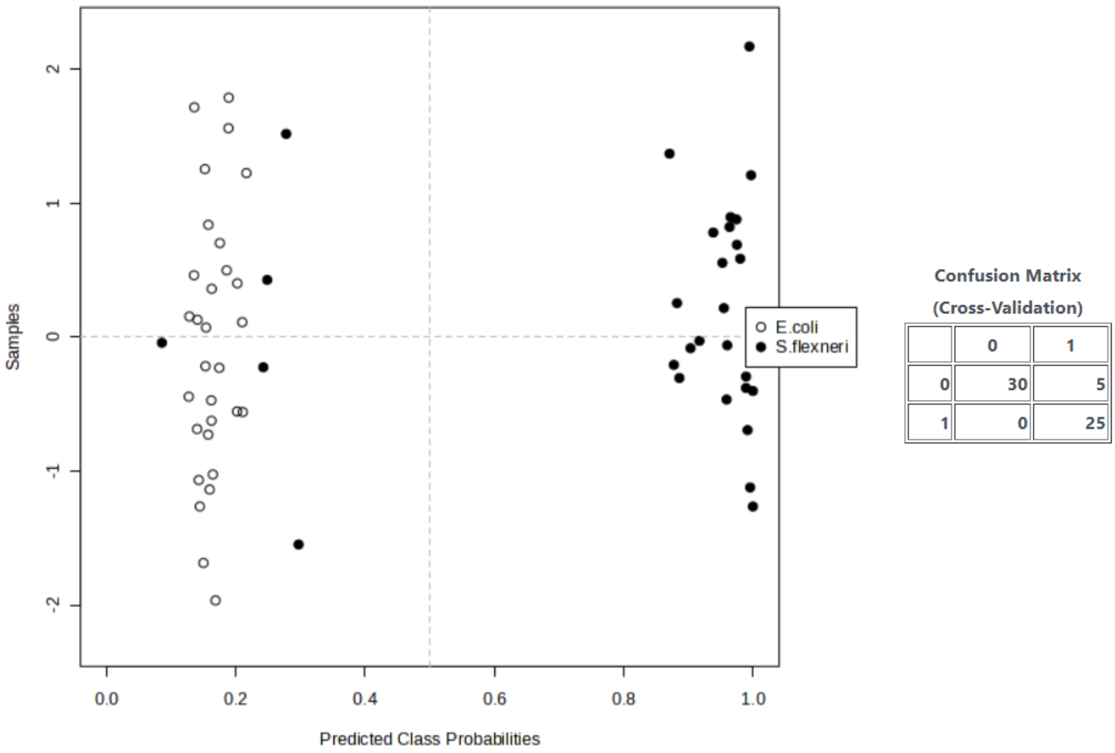

	0	1
0	30	5
1	0	25

Figure S5. Predicted class probabilities for *m/z* 175.126 obtained for Au-SALDI MS data from *E. coli* and *S. flexneri* metabolite extracts.

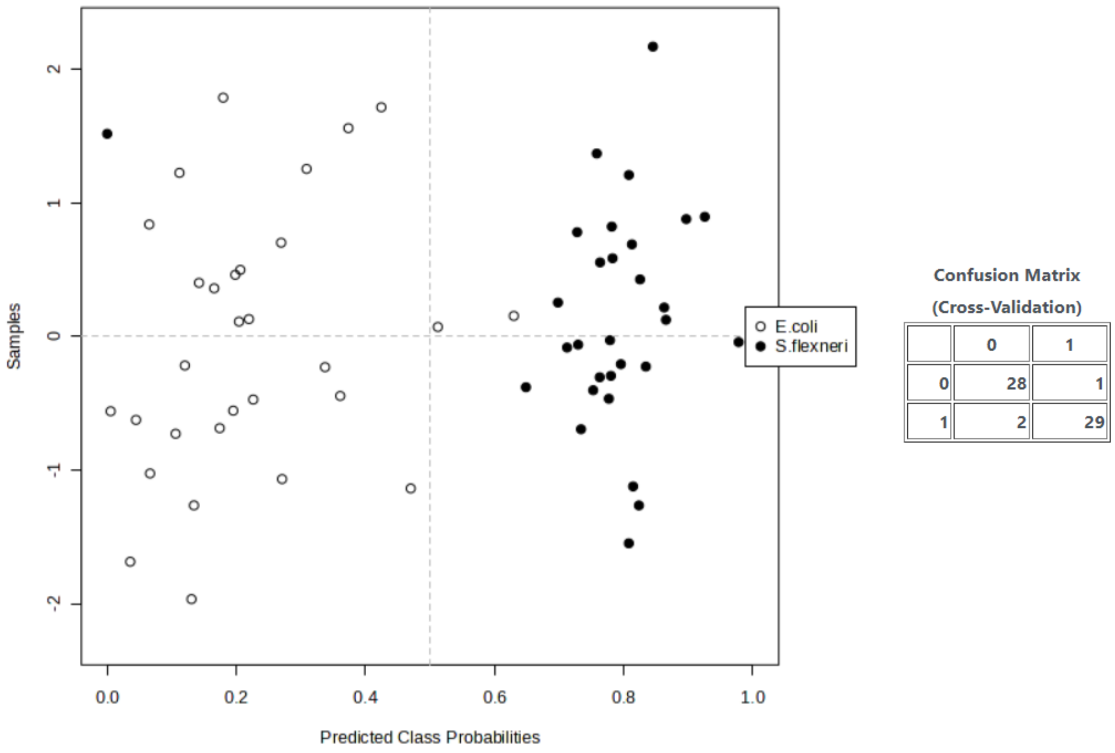

	0	1
0	28	1
1	2	29

Figure S6. Predicted class probabilities for *m/z* 181.015 obtained for Au-SALDI MS data from *E. coli* and *S. flexneri* metabolite extracts.

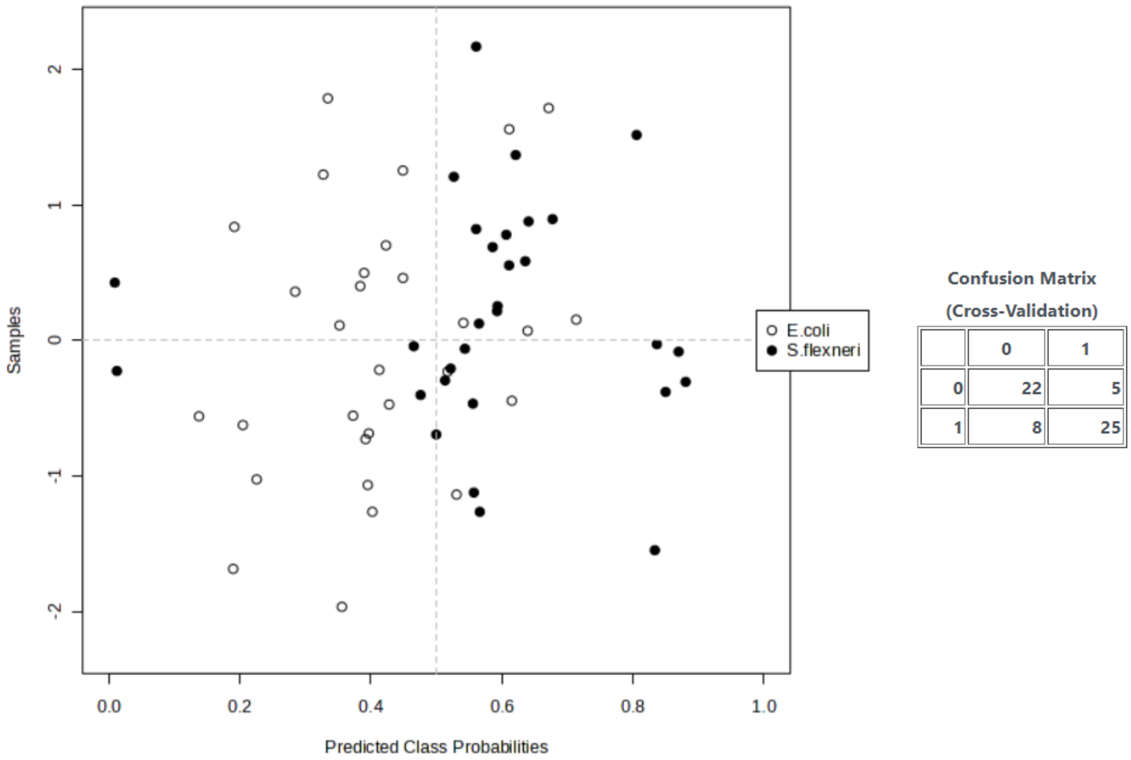

Figure S7. Predicted class probabilities for *m/z* 203.003 obtained for Au-SALDI MS data from *E. coli* and *S. flexneri* metabolite extracts.

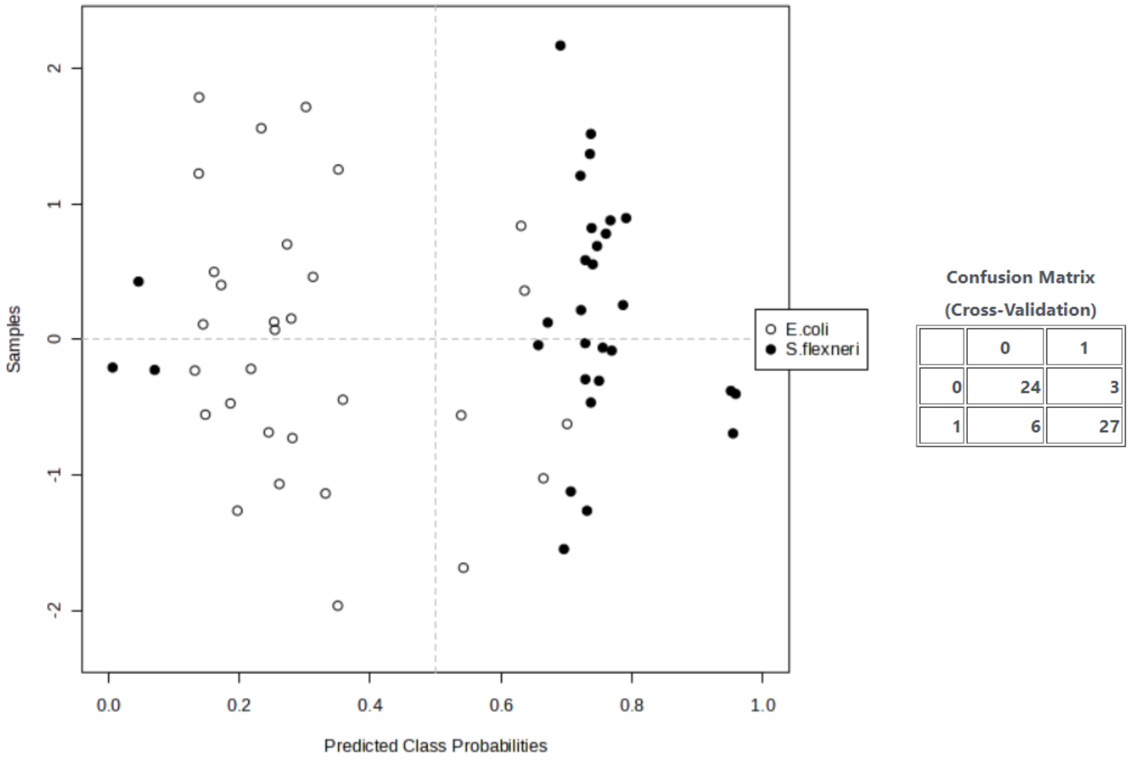

Figure S8. Predicted class probabilities for *m/z* 210.174 obtained for Au-SALDI MS data from *E. coli* and *S. flexneri* metabolite extracts.

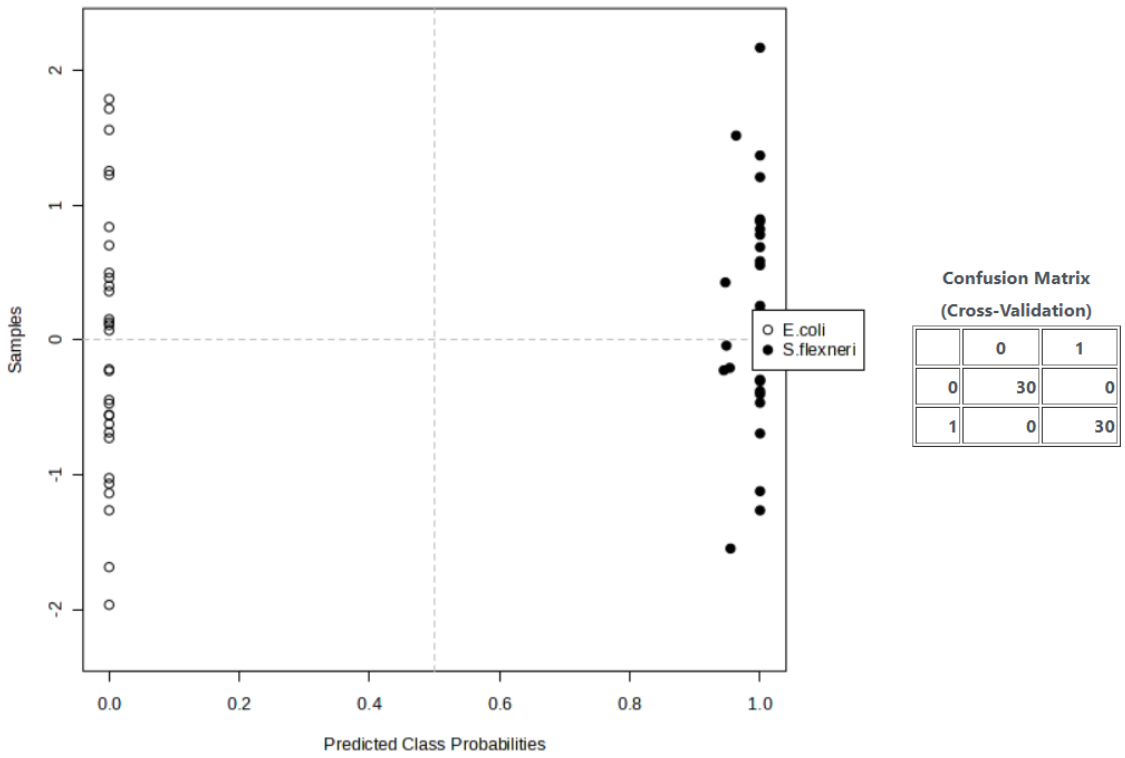

Figure S9. Predicted class probabilities for *m/z* 219.093 obtained for Au-SALDI MS data from *E. coli* and *S. flexneri* metabolite extracts.

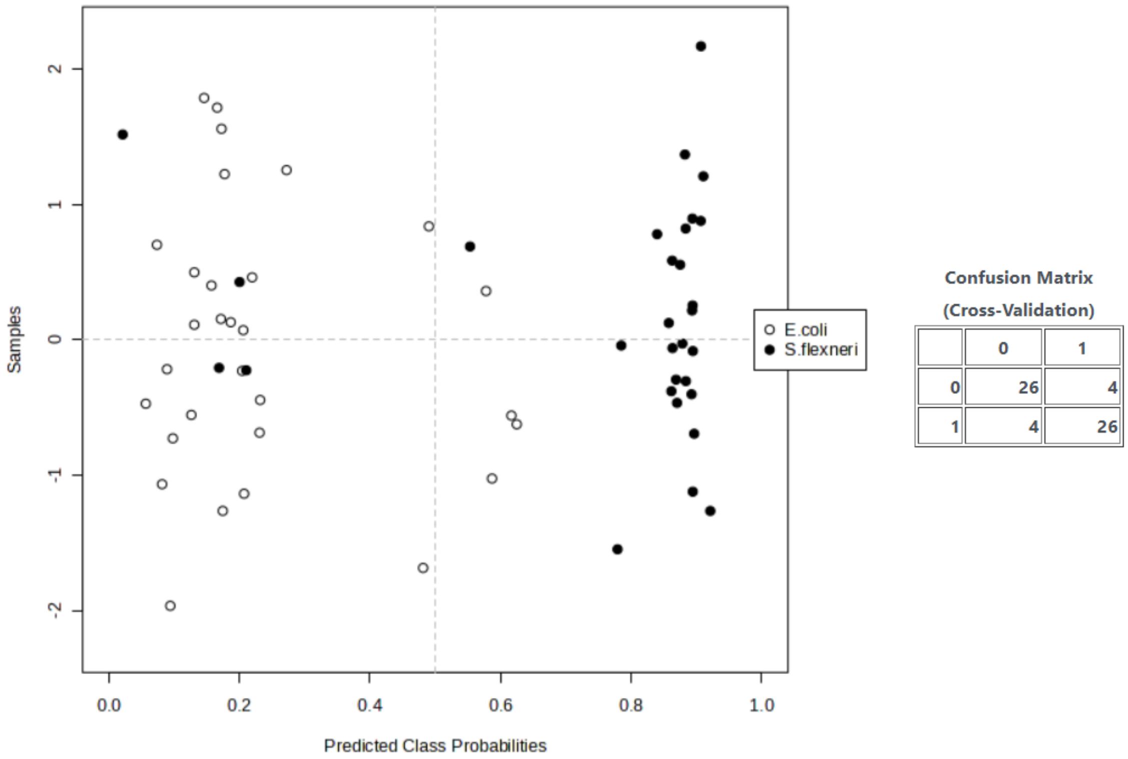

Figure S10. Predicted class probabilities for *m/z* 222.177 obtained for Au-SALDI MS data from *E. coli* and *S. flexneri* metabolite extracts.

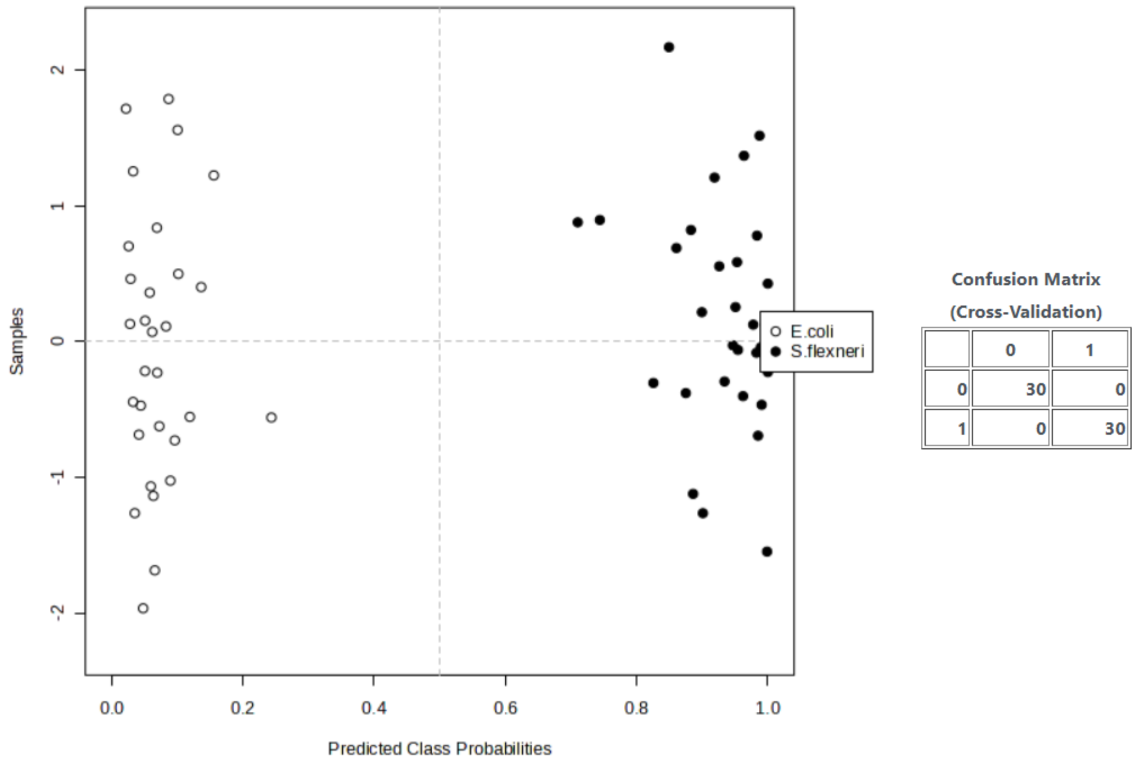

Figure S11. Predicted class probabilities for *m/z* 226.002 obtained for Au-SALDI MS data from *E. coli* and *S. flexneri* metabolite extracts.

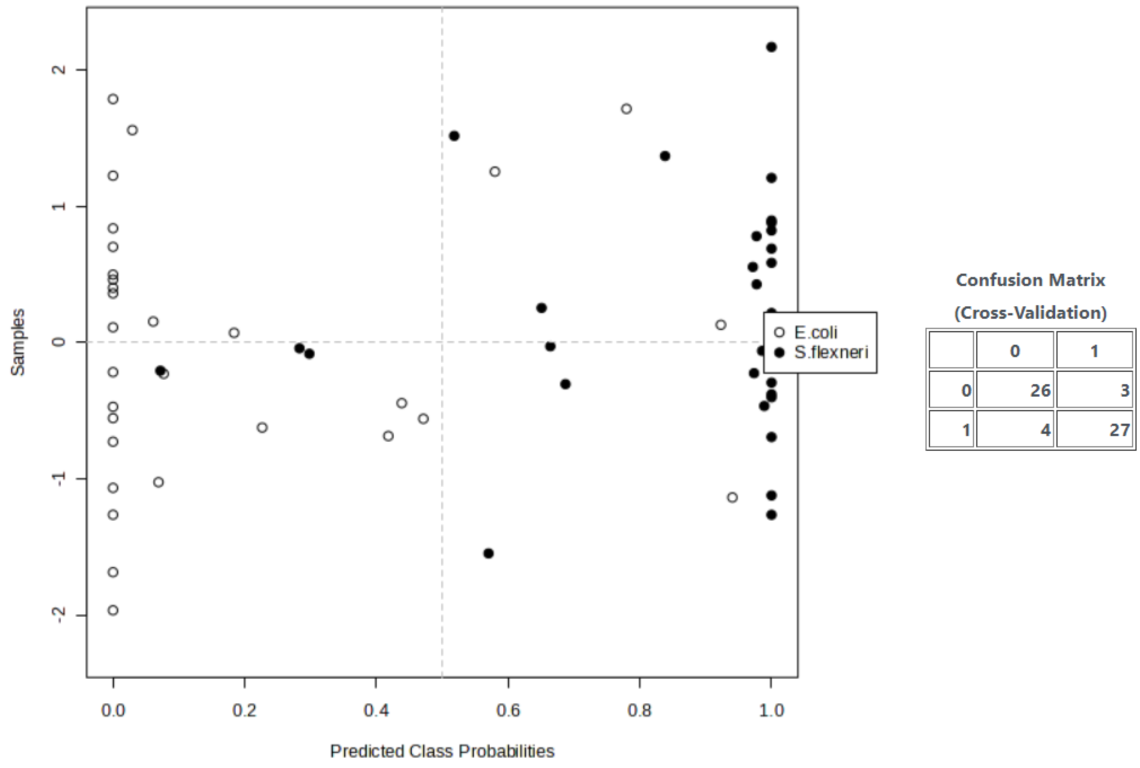

Figure S12. Predicted class probabilities for *m/z* 343.093 obtained for Au-SALDI MS data from *E. coli* and *S. flexneri* metabolite extracts.
